# Epigenetics and Expression of the Wnt Signaling Pathway in Ulcerative Colitis

**DOI:** 10.5152/tjg.2025.24619

**Published:** 2025-06-16

**Authors:** Zuhal Altintas, Mehmet Emin Erdal, Engin Altintas

**Affiliations:** 1 Department of Medical Genetics, Mersin University Faculty of Medicine, Mersin, Türkiye; 2 Department of Medical Biology and Genetics, Mersin University Faculty of Medicine, Mersin, Türkiye; 3 Department of Gastroenterology, Mersin University Faculty of Medicine, Mersin, Türkiye

**Keywords:** DNA methylation, inflammatory bowel disease, ulcerative colitis, Wnt pathway

## Abstract

**Background/Aims:**

Secreted frizzled-related proteins (SFRPs) are antagonists that bind Wnt and inhibit signaling through this pathway. Secreted frizzled-related proteins are silenced by promoter methylation and cause hyperactivation of the Wnt pathway. In this study, the aim was to evaluate the relationship between methylation and expression of genes involved in the Wnt signaling pathway and the risk of cancer development in inflammatory bowel disease.

**Materials and Methods:**

The patient group consisted of 20 individuals who were diagnosed with left-side ulcerative colitis and underwent surveillance colonoscopy; the control group consisted of 15 individuals without symptoms and endoscopic pathology who were screened for colorectal cancer. Tissue samples were obtained from inflamed and non-inflamed areas of the colon. Methylation and gene expression profiles of the Wnt pathway genes *APC1A, APC2, SFRP1, SFRP2, SFRP4,* and *SFRP5* were analyzed from DNA and RNA obtained from these tissues.

**Results:**

A significant correlation was found between the methylation status and expression of the *SFRP4* gene in the proximal colon in the patient group compared to controls (*P* = .018). For the methylation of the *APC2 *gene, 8 patients were methylated (40%), and 12 were unmethylated (60%), while 1 of the controls was methylated (6.7%) and 14 were unmethylated (93.3%) (*P* = .018). There was no statistically significant association between methylation, expression, and inflammation status for other genes between patients and controls.

**Conclusion:**

In ulcerative colitis, inflammation is thought to be associated with both increased *APC2* methylation and decreased expression findings due to decreased *SFRP4* methylation in non-inflamed areas. However, more research is needed to establish a link with ulcerative colitis-related neoplasia.

**Main Points**

- Methylation frequency of Wnt signaling pathway genes is increasing during the development of inflammatory bowel disease–associated neoplasia.
- Further research is required to show that the findings of increased methylation of *APC2*, decreased methylation of *SFRP4,* and decreased expression due to decreased methylation of *SFRP4 *are associated with ulcerative colitis-related neoplasia.
- Supporting the current findings with immunohistochemical studies will further increase the value of the findings.
- Further research is needed to identify precancerous dysplasia in UC patients, focusing on disease-specific methylation changes and mechanisms of gene expression regulation.
- This study proved the presence of histopathological inflammation in the endoscopically non-inflamed colon region (proximal colon).

## Introduction

Chronic and recurrent inflammatory disorders of unknown cause affecting the digestive system are known as inflammatory bowel diseases (IBD). Ulcerative colitis (UC) is a putative risk factor for the development of colorectal cancer (CRC), and the risk of developing CRC is 2 to 5 times higher in patients with ulcerative colitis than in the general population. Risk factors include chronic inflammation, long disease duration, younger age at diagnosis, presence of disseminated colitis, family history of a first-degree relative, genetic factors, coexisting primary sclerosing cholangitis, and acquired factors.^1^

Carcinomas associated with IBD develop in areas of dysplasia and flat areas that are difficult to detect during colonoscopy. A study reported that maternal methyl-diet supplementation increased offspring colitis susceptibility, which is associated with persistent epigenetic and long-term microbiome alterations.^2^ In the first epigenetic studies, DNA hypermethylation was reported to be the main epigenetic mechanism of colitis-associated CRC.^3^ In IBD, increased DNA methylation is observed in colonic epithelial cells due to inflammation and high cell turnover. Furthermore, increased DNA methylation has been shown to be more common in dysplastic colonic tissues compared to those without dysplasia.^4^

One of the events detected early in IBD colitis patients is the methylation of Wnt signaling genes. In the last 40 years of research, it has been elucidated how Wnt/β-catenin-mediated control, which is crucial for embryonic development, tissue regeneration capacity, and the initiation and spread of cancer, affects cell migration, differentiation, and proliferation.^6^ Wnt-induced cancers involve genetic alterations in Wnt pathway components as well as various epigenetic alterations associated with tumor initiation and progression.^7^

Wnt signaling dysregulation is one of the main causes of tumor growth and involves a sophisticated protein network that controls the regulation of molecular processes detected in many malignancies.^8^ The majority of methylation in the human genome occurs at CpG sites in gene promoters, accounting for around 1.5% of the total methylation. Many types of cancer are associated with abnormal hypermethylation at CpG (Areas rich in guanine and cytosine in DNA promoter regions are called CpG islands) sites.^9^ Extracellular Wnt signaling inhibitors known as secreted frizzled-related proteins (*SFRPs*) work by attaching themselves to Frizzled receptors or Wnt ligands directly. Secreted frizzled-related proteins are silenced by promoter methylation in CRC and cause hyperactivation of the Wnt pathway.^10^ Suzuki et al^11^ reported that SFRP2, SFRP4, and SFRP5 are suppressed via promoter hypermethylation in CRC, which may lead to the down-regulation of mRNA and protein synthesis.

Adenomatous polyposis coli (APC) is an important component that functions as a scaffold in the β-catenin degradation complex within the Wnt signaling pathway. It is encoded by 2 distinct genes, APC1 and APC2.^12^ Ahmet et al^13^ reported that APC proteins are necessary to control Wnt signaling in all cells and that their combined activities provide tight control over β-catenin-mediated transcriptional activation. Hypermethylation of the APC1A and APC2 promoters in CRC has been reported to accompany the loss of expression of its transcript.^14^ ^15^

In colorectal and breast cancer, promoter hypermethylation has been shown to silence SFRP 1, 2, 4, and 5 and lead to abnormal Wnt signaling activation. In these patients, promoter hypermethylation was consistent with cancer expression and stage of malignancy.^11^ ^14^^16,17^

The aim of this study is to evaluate the relationship between the methylation and expression of *APC, SFRP1, SFRP2, SFRP4*, and *SFRP*5 genes involved in the Wnt signaling pathway and the risk of cancer development in IBD.

## Materials and Methods

Patients with left-sided ulcerative colitis who were clinically in remission and undergoing surveillance were included in the study to ensure patient homogenization and to see if there was a difference between the inflamed and non-inflamed areas. The patient group used only oral mesalazine. The patient group consisted of 20 patients. Informed consent was obtained from all participants. Ethics committee approval for this study was obtained from Mersin University Local Ethics Committee with decision no: 63 dated April 17, 2009.

In patients with UC (ulcerative colitis) who underwent surveillance colonoscopy, it started from the cecum and was inspected every 10 cm. Two biopsy samples were taken from each of the 4 quadrants every 10 cm up to the anal canal. If malignancy or a dysplasia-associated lesion-mass was suspected in the patient group, an additional biopsy was taken. Biopsies were taken from both normal and inflamed colonic mucosa of patients with left-sided UC and investigated whether there was a difference in the methylation and expression of Wnt pathway genes studied between inflamed and non-inflamed mucosa in UC patients. In control group patients, biopsies were taken from the 20th centimeter of the rectum and 10 cm from the cecum in the same manner as in surveillance colonoscopies.

The Rachmilewitz Endoscopic Score was performed for endoscopic assessments. Endoscopic remission was defined as an endoscopic index score of 0-4 points.^18^ Histological activity was graded as no inflammation, mild, or severe. The presence of architectural changes without changes in the density and composition of the cellular infiltrate of the lamina propria means “no inflammation.” Mild inflammation is defined by the presence of architectural changes (irregular surface and crypt abnormalities) and an increase in lamina propria mononuclear cells. Severe inflammation is defined by the presence of neutrophils in conjunction with epithelial cell damage.

The quantitation of *SFRP2, SFRP4, SFRP5, APC1*,* and APC2* genes was determined by performing “Comparative CT (ΔΔCt)” analysis by Real-Time Polymerase Chain Reaction (RT-PCR). The human Beta-actin gene (ACTB) was used as a control gene. In this study, methylation transformations were performed on DNA obtained from patients and controls using the EZ DNA Methylation-Gold™ kit according to the manufacturer’s recommendation.

### Statistical Analysis

Independent sample *t*-test was used in the comparisons of ulcerative colitis patients and control groups in terms of age and expression levels and in the comparisons of expression levels in patients according to methylation groups. Chi-square or Likelihood ratio test was used to analyze categorical variables. Statistical analyses were performed with the SPSS v.11.5 package program (SPSS Inc.; Chicago, IL, USA). Results were considered significant if *P* < .05 in statistical analysis.

## Results

a. **Patient: **The patient group consisted of 20 individuals diagnosed with left-sided UC. The control group consisted of 15 individuals without any complaints and endoscopic pathology who were screened for colon cancer. In the UC patient group, 16 out of 20 individuals were male (80%), and 4 were female (20%); in the control group, 7 out of 15 individuals were male (46.7%), and 8 were female (53.3%). The median disease duration of patients diagnosed with left-sided UC was 13.6 years. In a patient with UC, a 1-centimeter polyp was observed in the proximal part of the ascending colon. Histopathologic examination of the excised polyp material revealed low-grade dysplasia. However, since this patient did not come for follow-up afterward, his medical records could not be accessed. On histopathologic examination, 13 patients had severe active inflammation, and 7 had mild inflammation in biopsy specimens taken from the left-side colon. In biopsy specimens taken from the proximal colon, 12 patients had mild active inflammation and 8 had no inflammation. Of the 8 patients without inflammation in the proximal colon, 4 had mild active inflammation in the left-side colon and the other 4 had severe active inflammation. The clinical and inflammatory characteristics of the patients are summarized in Table 1. Unfortunately, the effect of smoking could not be evaluated because smoking history could not be obtained from the medical records of the patient and control groups.

The mean age of the patients with ulcerative colitis (n = 20) was 53.5 ± 12.9 years and 53.6 ± 9.8 years in the control group (n = 15), and this difference was not statistically significant (*P* = .967).

b. **Methylation: **There was a statistically significant difference between patients and controls in terms of methylation of the *SFRP*4 gene in the proximal colon (*P* = .018). There were no statistically significant differences between patients and controls in terms of methylation of *SFRP*2, *SFRP*5, *APC*1, and *APC*2 genes in the proximal colon, respectively (*P* = .431, *P* = .486, *P* = .486, *P* = .235). In the proximal colon samples of the patients, methylation was reduced compared to controls. There was a statistically significant difference between patients and controls in terms of methylation of the *APC*2 gene in the left side colon (*P* = .018). There were no statistically significant differences between patients and controls in terms of methylation of *SFRP*2, *SFRP4, SFRP5,* and *APC1* genes in the left side colon, respectively (*P* = .385, *P* = .834, *P* = .433, *P* = .093). Methylation of the *APC*2 gene was increased in the left side colon of patients compared to controls. The left side and proximal colon methylated and unmethylated frequencies of patients and controls are summarized in Table 2.
c. **Expression:** Samples from the patient and control groups’ proximal and left-side colons showed no differences in the expression levels of the *SFRP2*, *SFRP4*, *SFRP5*, *APC1*, and *APC2* genes. No significant correlation was found between the inflammation status (mild/severe) and the expression levels of *SFRP2, SFRP4, SFRP5,* *APC*1, and *APC*2 genes in the left-side colon (Table 3).

## Discussion

In this study, the methylation status and expression levels of the genes *SFRP2, SFRP4, SFRP5,* *APC*1, and *APC2* genes, which are involved in the Wnt pathway, were investigated in samples taken from the proximal and left side colon of patients with left-sided UC who underwent surveillance colonoscopy, and healthy individuals who underwent cancer screening. In the proximal colon, there was a statistically significant difference in the methylation of the *SFRP*4 gene between patients and controls (*P* = .018).

While most colorectal tumors show activation of the Wnt/β-catenin pathway, the course of the disease varies from person to person even at the same tumor stage, and this heterogeneity is determined by the genetic composition of each tumor and the mix of mutations caused by cancer.^19^ *SFRP*s can act as anti-Wnt agents and inhibit the effect of Wnt ligands on cancer growth. The abnormal methylation of *SFRP* and the loss of *SFRP* gene expression in various types of human cancers initiate the activation of the Wnt pathway, an important mechanism for tumor formation and development, leading to the hyperactivation of the Wnt pathway. This hyperactivation can promote cell proliferation, survival, and metastasis, ultimately contributing to tumor progression. Understanding the regulatory role of SFRPs in the Wnt signaling pathway may offer new therapeutic targets for inhibiting cancer growth. It has been shown that the risk of CRC is significantly associated with the hypermethylation of SFRP1, SFRP2, SFRP4, and SFRP5.^16^ Dhir and colleagues reported that the frequency of methylation of genes in the Wnt signaling pathways gradually increased from normal colon to IBD colitis and IBD-associated neoplasia.^5^ In this study, there was a statistically significant decrease in SFRP4 gene methylation in the proximal colon samples of patients when compared to controls (*P* = .018).

The *APC* gene, a tumor suppressor gene, plays an important role in the development of CRC by reducing or eliminating cell adhesion functions and promoting the infiltration and metastasis of cancer cells.^14^ According to a meta-analysis study by Liang and colleagues, there was a significant relationship between the risk of CRC incidence and the frequency of APC promoter hypermethylation, and this frequency was significantly higher in adenoma tissues compared to normal colorectal tissues.^20^ He et al^15^ reported that APC2 hypermethylation levels are significantly higher in female patients compared to male patients and that APC2 in colorectal tissues is significantly hypermethylated in tumors compared to surrounding tissues. According to the results of module 1 of the ENDCAP-C study published in 2018, a panel of 5 markers (SFRP2, SFRP4, WIF1, APC1A, and APC2) used to identify neoplastic mucosa was reported to have excellent diagnostic accuracy in detecting precancerous and invasive neoplasia.^21^ According to the results of the ENDCaP-C diagnostic accuracy study, patients who underwent an initial colonoscopy were subjected to a methylation test. Subsequently, patients without neoplasia underwent a second (reference) colonoscopy and methylation. According to the second test, the number of patients with neoplasia associated with primary methylation changes increased, but this difference was not statistically significant. Based on this study, it has been reported that methylation testing cannot be recommended for patients with chronic ulcerative colitis.^22^

In a study investigating prognostic factors in CRC, He et al^15^ reported that APC2 is hypermethylated and may serve as a biomarker of tumor formation in Chinese CRC patients.

In this study, it was found that methylation of the *APC*2 gene was increased in the left-side colon of patients compared to controls, and this was statistically significant (*P* = .018). The finding was consistent with the findings of He et al.^15^ In this study, no correlation was found between the expression levels of the SFRP2, SFRP4, SFRP5, APC1, and APC2 genes in the proximal and left colon samples taken from the patient and control groups. Aust et al^23^ reported that *APC* expression is reduced in UC-associated cancers and sporadic CRCS compared to normal epithelium. In this study, there was no difference in the expression levels of the *APC*1 and *APC*2 genes in samples taken from the proximal and left-sided colon of the patient and control groups. In this study, there was no difference in the expression levels of the APC1 and APC2 genes in samples taken from the proximal and left colon of the patient and control groups.

Recent studies have revealed conflicting data regarding the expression level and role of SFRP4 in various malignancies, unlike other members of the SFRP family.^24^ In most cancers, it was found that SFRP4 expression is either reduced or suppressed. SFRP4 is overexpressed in CRC and has shown less frequent promoter hypermethylation in tumors compared to normal mucosa, suggesting that it may function as an oncogene.

For the *SFRP5* gene, a statistically significant correlation was found between the methylation status and expression levels of the samples taken from the distal colon of the patients (*P* = .015), and it was observed that as the methylation of the SFRP5 gene in the distal colon increased, its expression decreased.

Zhou et al^25^ reported that the suppression of *SFRP2* inhibited CRC cell proliferation, migration, and invasion and that CRC patients with high *SFRP2 *expression had shorter overall survival times, recommending that *SFRP2* could be a major prognostic marker in CRC. In the patient group, the SFRP2 gene expression level in distal colon samples was found to be 1.39 ± 0.42, and in the control group to be 1.14 ± 0.32. However, there was no correlation between the SFRP2 gene expression levels in the proximal and distal colon in both the patient and control groups (*P* = .07).

Dhir et al^5^ reported that the Wnt signaling pathway genes are an early event in patients with IBD colitis and gradually increase as IBD-associated neoplasia develops. Additionally, they indicated that stool-based DNA methylation methods could be developed for the early identification of cancer and dysplasia in IBD patients using the methylation of APC1A, APC2, SFRP1, and SFRP2.^5^

Zhang et al^26^ have provided detailed evidence that smoking has a definitive relationship with the risk of IBD and that epigenetic changes play a key role in this relationship. These results provide evidence that epigenetic changes are associated with IBD and support the thesis. However, the patients’ smoking status was not examined in this study. Kim et al^27^ demonstrated in their study on metastatic or recurrent CRC patients that increased methylation of 4 Wnt pathway genes are associated with worse clinical outcomes. They have reported that this finding could lead to more personalized treatment planning for early-stage CRC patients and better clinical outcomes in the future. It has been reported that the use of NSAIDs reduces the risk of CRC by 40%-50% and has a positive effect on advanced CRC. NSAIDs may also inhibit Wnt signaling, thereby blocking the tumor-promoting pathway.^28^

However, there is insufficient evidence to suggest that they can be used to prevent CRC in patients with IBD.^29^

In this study, the methylation and expression status of SFRP2, SFRP4, SFRP5, APC1, and APC2 genes were evaluated for the first time in ulcerative colitis patients in the Turkish population. After analyzing all of the available information, it was proposed that diagnosis and therapy planning for IBD-associated neoplasia may benefit greatly from high methylation levels of Wnt signaling pathway genes during the disease’s progression. In this study, the small sample size was one of the disadvantages that might have limited the statistical power. Another limitation is that only mRNA levels were analyzed in the study, and validation at the protein level was not performed using techniques such as Western blotting or immunohistochemistry. Additionally, covariates that could affect DNA methylation status were not analyzed.

## Figures and Tables

**Table 1.** Clinical and Inflammatory Characteristics of the Patients

Patient	PMI	DMI	PEI	DEI	Disease Duration	Age	Gender
UK-1	N/A	S	None	S	15	41	M
UK-2	N/A	S	None	MI	18	46	M
UK-3	MI	S	None	S	14	61	F
UK-4	N/A	S	None	S	12	36	F
UK-5	MI	S	None	S	12	33	M
UK-6	MI	MI	None	MI	15	59	M
UK-7	MI	S	None	MI	10	37	M
UK-8	MI	MI	None	S	15	47	M
UK-9*	N/A	S	None	S	11	69	M
UK-10	N/A	MI	None	MI	15	56	M
UK-11	MI	MI	None	MI	10	40	M
UK-12	N/A	MI	None	S	13	49	M
UK-13	N/A	MI	None	S	12	68	M
UK-14	MI	S	None	S	16	62	M
UK-15	MI	S	None	S	17	67	M
UK-16	MI	S	None	S	13	80	M
UK-17	MI	S	None	MI	11	67	F
UK-18	MI	S	None	MI	10	53	M
UK-19	MI	S	None	S	15	49	F
UK-20	N/A	MI	None	S	19	50	M

DEI, distal endoscopic inflammation; DMI, distal microscopic inflammation; F, female; M, male; MI, mild inflammation; PEI, proximal endoscopic inflammation; PMI, proximal microscopic inflammation; S, severe inflammation.

*The patient who is detected polyp.

**Table 2.** Left Side and Proximal Colon Methylation and Unmethylation Frequencies of Patients and Controls

Gene	Left Side					Proksimal				
	Methylated (n/%)		Unmethylated (n/%)		*P*	Methylated (n/%)		Unmethylated (n/%)		*P*
	Patient	Control	Patient	Control		Patient	Control	Patient	Control	
*SFRP2*	19/95	13/86.7	1/5	2/13.3	.385	17/85	14/93.3	3/15	1/6.7	.431
*SFRP4*	19/95	14/93.3	1/5	1/6.7	.834	12/60	14/93.3	8/40	1/6.7	.018
*SFRP5*	12/60	7/46.7	8/40	6/53.3	.433	13/65	8/53.3	7/35	7/46.7	.486
*APC1*	4/20	7/46.7	16/80	8/53.3	.093	11/55	10/66.7	9/45	5/33.3	.486
*APC2*	8/40	1/6.7	12/60	14/93.3	.018	6/30	2/13.3	14/70	13/86.7	.235

**Table 3.** Means and SDs of Inflammation and Expression Ratio (E) of *SFRP2*, *SFRP4*, *SFRP5*, *APC1*, and *APC2* Genes in Left-Side Colon Samples of Patients

Gene	Inflammation Status	N	Mean	SD	Standard Error	*P*
*APC1*	M	7	3.39000	3.559290	1.345285	.771
	S	12	2.84108	4.084111	1.178981	
*APC2*	M	7	1.29314	0.484805	0.183239	.828
	S	11	1.37691	0.921628	0.277881	
*SFRP2*	M	7	1.56800	0.487312	0.184187	.180
	S	13	1.29646	0.373188	0.103504	
*SFRP4*	M	7	1.33357	0.421877	0.159455	.278
	S	13	1.09500	0.470273	0.130430	
*SFRP5*	M	7	1.09214	0.632811	0.239180	.832
	S	13	1.14254	0.419344	0.116305	

M, mild; S, severe.

## Data Availability

The data that support the findings of this study are available on request from the corresponding author.
